# Is There Difference between the Effects of Two-Dose Stimulation for Knee Osteoarthritis in the Treatment of Heat-Sensitive Moxibustion?

**DOI:** 10.1155/2012/696498

**Published:** 2012-06-07

**Authors:** Rixin Chen, Mingren Chen, Jun Xiong, Zhenhai Chi, Meiqi Zhou, Tongsheng Su, Jianhua Sun, Fan Yi, Bo Zhang

**Affiliations:** ^1^Acupuncture and Rehabilitation Department, The Affiliated Hospital, Jiangxi University of TCM, 445 Bayi Avenue, Nanchang 330006, China; ^2^Acupuncture Department, The First Affiliated Hospital, Anhui University of TCM, Hefei 330006, China; ^3^Acupuncture Department, Shanxi TCM Hospital, Xian 330006, China; ^4^Acupuncture Department, Jiangsu TCM Hospital, Nanjing 330006, China

## Abstract

Considering that the dosage of manipulating Moxa plays an important role in obtaining good effects for heat-sensitive moxibustion, it would be valuable to know whether the use of fixed dosage is as effective as the use of an individual one. The paper carried out a rigorous multi-centre randomized controlled trial, and its result demonstrated that the effectiveness of individual eliminate-sensitive dosing regimen might more superior to the stable conventional dosing regimen in the treatment of KOA. According the record of individual moxibustion time, the dosage differed in the terms of patients' conditions and moxibustion sensation, which had been measured about 47.30 ± 6.20 minutes (28 ~ 65 minutes).

## 1. Background

Suspended moxibustion is a traditional Chinese medical intervention that involves the burning of moxa indirectly at the acupoints. Moxibustion has anti-inflammatory or immunomodulatory effects against chronic inflammatory conditions in humans [1]. Heat-sensitive moxibustion therapy is one of common suspended moxibustion treatments in China [2]. A great deal of physicians utilized heat-sensitive moxibustion therapy in different kinds of diseases in China. Moreover, several articles and research reports have reported the effectiveness and safety of heat-sensitive moxibustion for KOA [3–8].

Moxibustion uses heat stimulation at various temperature levels from mild skin warming to tissue damage from burning. Therefore, the dose of manipulating Moxa plays an important role in obtaining good effects. The regimen of moxibustion used for treatment of KOA has varied between studies.

To sum up, two main regimens were carried out in these papers. One selected the stable conventional dose, which was recommended by the universal text book [1]. The therapy was executed for 15 minutes per acupoint. The other one held that the dose differed in the terms of patients' conditions and moxibustion sensation. Treatment sessions should be ended when patients felt the acupoint heat-sensitization phenomenon disappeared. We called it individual eliminate-sensitive dose. Therefore, it would be valuable to know whether there is difference between the effects of two-dose stimulation for KOA in the treatment of heat-sensitive moxibustion. We planned the rigorous multicentre randomized controlled trial, in order to seek for optimal dose of the best therapeutic effect.

## 2. Methods

### 2.1. Objective

The aim of this study is to compare the effectiveness of an individual eliminate-sensitive dose and a stable conventional dose in the treatment of patients with moderate to severe swelling KOA in China.

### 2.2. Sample Size

The sample size for testing the difference between means was calculated with the SPSS 13.0 programme, by setting the standard deviation of the guiding principle of clinical research on new drugs in the treatment of KOA (GPCRND-KOA) [9] score of 2.55 and 3.39 [5, 6] and mean difference of total GPCRND-KOA score between groups of 2.50, power of 0.9, and a level of significance at 0.05. Allowing for a 20% loss to follow up, a total of 36 participants were required in each group, with 72 participants in total.

### 2.3. Design

A multicentre (four centers in China), randomized, and assessor blinded, controlled trial was conducted at the Affiliated Hospital with Jiangxi University of Traditional Chinese Medicine (TCM) in Nanchang, The first Affiliated Hospital with Anhui University of TCM in Hefei, Jiangsu TCM Hospital in Nanjing, and Shanxi TCM Hospital in Xian. The study was sequentially conducted as follows: a run-in period of one week prior to randomization, a treatment period of 30 days, and a follow-up period of six months. At the end of the run-in period, participants were randomized to the individual eliminate-sensitive dose group or the stable conventional dose group by the central randomization system. This system was provided by China Academy of Chinese Medical Sciences, which adopted the computer telephone integration (CTI) technology to integrate computer, internet, and telecom. The random number list was assigned by interactive voice response (IVR) and interactive web response (IWR) [10]. The success of blinding was assessed at each participant's last visit. Data collection staff, and data analysts were blinded to treatment group.

### 2.4. Participants

#### 2.4.1. Recruitment

A total of 72 eligible patients were enrolled in the study between December 30, 2009, and March 18, 2010, from this multisite. The ethics committees of the Affiliated Hospital with Jiangxi University of TCM approved the study.

#### 2.4.2. Inclusion Criteria

Eligible participants were those previously diagnosed with moderate to severe swelling KOA, according to the GPCRND-KOA criteria (>5 score). Patients were required to complete the baseline KOA diary. Written informed consent was obtained from each participant. The inclusion criteria restricted the following conditions. According with the below KOA diagnosis standard, meanwhile, knee joints appeared swell; Floating patella test was negative; patients accepted the treatment protocol in this trial; acupoint heat-sensitization phenomenon existed in the region consisting of Yin Lingquan (SP9), Yang Lingquan (GB34), Liang Qiu (ST34), and Xue Hai (SP10). Patients had been stopped receiving previous intervention before recruitment for two weeks.

#### 2.4.3. Exclusion Criteria

Participants were excluded if they suffer from serious life-threatening disease, such as disease of the heart and brain blood vessels, liver, kidney, and hematopoietic system, and psychotic patients. We excluded patients with diabetes, diabetic polyneuropathy, and polyneuropathic disturbances. Participants were not being eligible if the females are in the duration of pregnancy or lactation. The following conditions were also excluded items: acute knee joint trauma or ulceration in its local skin, complicated with serious genu varus/valgus and flexion contraction.

### 2.5. Study Interventions

Qualified specialists of acupuncture in TCM with at least five years of clinical experience performed the moxibustion in this study. All treatment regimens were standardized between four centers practitioners via video, hands-on training, and internet workshops. Both groups of patients were requested not to receive other treatments including any physical therapies, any pain-killing medicines, and acupuncture treatment from another place.

In the two groups, 22 mm (diameter) × 120 mm (length) moxa-sticks (Jiangxi TCM Hospital, China) were used. The patient was usually in the comfortable supine position for treatment, with 24°C~30°C temperature in the room. He should be wearing loose trousers, especially making his knee joints exposed.

#### 2.5.1. The Individual Eliminate-Sensitive Dose Group

For the group, the moxa-sticks were lit by the therapist and held over the region consisting of Yin Lingquan (SP9), Yang Lingquan (GB34), Liang Qiu (ST34), and Xue Hai (SP10). The warming suspended moxibustion lied 3 cm far from the surface of skin was used to search the acupoint heat-sensitization phenomenon. The areas were brought mild warmth without burning by moxa-sticks and manipulated until the patient reported the characteristic heat sensitization sensation, said to indicate effective moxibustion, that is commonly called De Qi. Patients felt comfortable in the moxibustion manipulation.

The following patients sensation suggested the special heat sensitization acupoint: diathermanous sensation due to moxa-heat, defining as the heat sensation conducting from the moxa local skin surface into deep tissue, or even into the joint cavity; expand heat sensation due to moxa-heat, defining as the heat sensation spreading the surrounding little by little around the moxa point; transfer heat sensation due to moxa-heat, defining as the heat sensation transferring along some pathway or direction, even to the ankle or hip conduction. When some acupoint existed one below sensation at least, the therapists marked the point as heat-sensitive acupoint. We tried our best to seek all the special acupoints in each patient by the repeated manipulation.

After obtaining the heat sensitization sensation, the therapists began to treat patient's at these heat-sensitive acupoints. There was a therapist working on the patient for the whole time of moxibustion. For moxibustion manipulation, the intensity of the given stimulation was varied with heat-sensitive sensation. Treatment sessions ended when patients felt the acupoint heat-sensitization phenomenon disappeared.

In the course of manipulation, the therapist recorded the time length of every patient from the beginning to the end in per treatment session. Patients received the treatment two times/day in the 1st week (one time/day from the 2nd week) for a total of 35 sessions over 30 days.

#### 2.5.2. The Stable Conventional Dose Group

Common practices were similar with the first group. Only one difference was that patients in this group received the identical dose (15 minutes).

### 2.6. Outcome Measures

Ministry of Health of the People's Republic of China (MHPRC) has proposed the guiding principle of clinical research on new drugs (GPCRND) [9]. In this criteria, a patient with KOA was assessed including pain, the relation between activity and pain, function impairment, and special exams. This scoring system was previously validated. The degree of KOA is divided into three levels: mild—<5 scores; moderate—5~9 scores; severe—>9 scores. In the terms of swelling knee, knee circumference was assessed at each time point. The parameter was measured in centimeters across the middle of a patella, with ordinary tape measure [11].

Therapeutic effect was assessed by comparing baseline and final conditions reported by the patient. This trial also recorded adverse effects reported by patients during treatment. The outcome measures above were assessed before the treatment (month 0), at the end of the treatment period (month 1), and 6 months after the end of the treatment period (month 7).

### 2.7. Statistical Methods

We conducted analysis on an intention-to-treat basis, including all randomized participants with at least one measurable outcome report. Analyses will be conducted using 2-sided significance tests at the 5% significance level. The statistician conducting the analyses remained blind to treatment group and data was only unblended once all data summarized and analyses were completed. All analyses were conducted in the SAS statistical package program (ver.9.1.3).

Baseline characteristics were shown as mean standard deviation (SD) for continuous data including age, previous duration, and others. As for participants' gender, *n* (%) of male and female in each group were shown as baseline characteristics. We will consider *P* < 0.05 as statistically significant.

Outcome measures were summarized descriptively (mean, SD, median, minimum, and maximum) at each time point by treatment group. The *t*-test, Mann-Whitney *U*, and Wilcoxon test were used for comparison of variables, as appropriate. All adverse events reported during the study were included in the case report forms; the incidence of adverse events were calculated. Missing data was replaced according to the principle of the last observation carried forward.

### 2.8. Adverse Events

We defined adverse events as unfavorable or unintended signs, symptoms, or disease occurring after treatment that were not necessarily related to the moxibustion intervention.

### 2.9. Ethics

Written consent was obtained from each participant. The study was conducted by a coordination center at the Affiliated Hospital with Jiangxi University of TCM in Nanchang and was approved by the Ethics Committee of the Affiliated Hospital of Jiangxi University of TCM.

## 3. Results

### 3.1. Recruitment and Baseline

**Figure 1:**
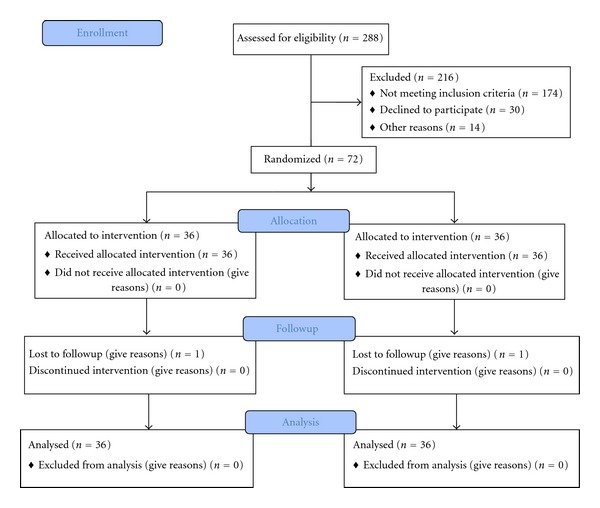
Flow diagram.

Participants 42~70 years of age were recruited from outpatient and inpatient in four centers. Of 288 screened patients, 216 could not be included in the study, mainly because they did not meet all eligibility criteria (Figure 1). Thus, 72 patients were randomly assigned into 2 treatment groups by 30 investigators. In follow-up visit, two patients dropped out of the study. One was in the individual eliminate-sensitive dose: because of falling injury of Legs. One patient in the stable conventional dose group was lost because of requiring another treatment in other hospitals. We also questioned patients about the intake of medication. Of 34 patients receiving pharmaceutical intervention before recruitment, 20 took NSAIDS such as ibuprofen, and Naprosyn; 14 were treated by glucosamine/chondroitin. There were no pretreatment differences among the two treatment groups at baseline (Table 1).

### 3.2. Outcome

#### 3.2.1. Total GPCRND-KOA Score

**Figure 2:**
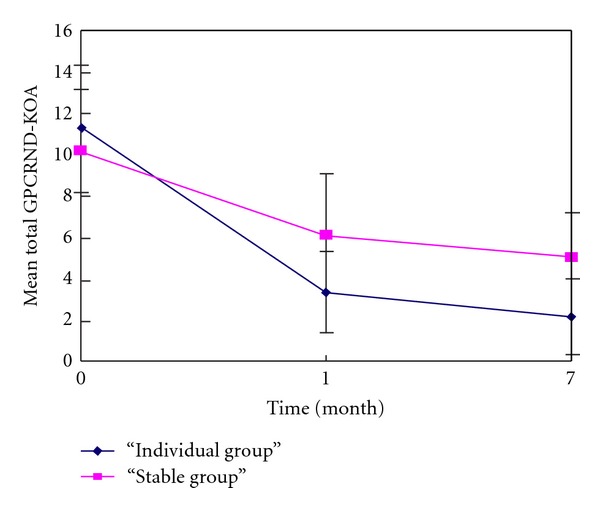
Mean total GPCRND-KOA (ITT, intention to treat analysis) at each time point.

Reductions in mean total GPCRND-KOA score at months 1 and 7 compared to baseline were observed and were highly significant (*P* < 0.001 for all comparisons). At both time points, there was highly significant difference between the groups (*P* < 0.01) as shown in Table 2.  Figure 2 shows that the mean total GPCRND-KOA score of both groups reduced sharply at the first month then remained decreasing to the end of month 7.

#### 3.2.2. Knee Circumference

**Figure 3:**
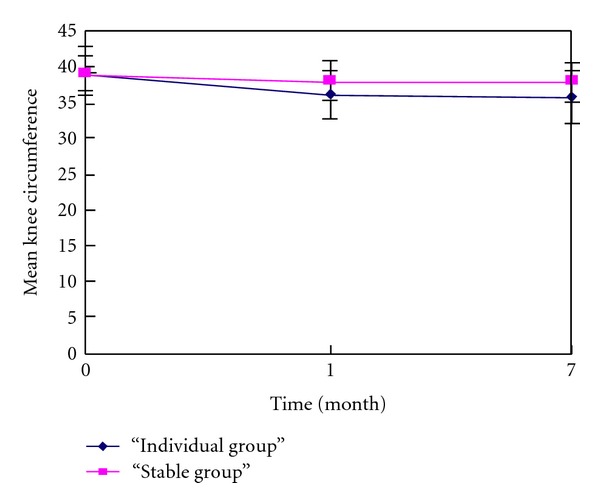
Mean knee circumference (ITT, intention to treat analysis) at each time point.

Reductions in mean knee circumference at months 1 and 7 compared to baseline were observed and were significant (*P* < 0.05 for all comparisons). At both time points, there was significant difference between the groups (*P* < 0.05) as shown in Table 3.  Figure 3 shows that the mean knee circumference of both groups reduced a few at the first month then remained decreasing to the end of month 7.

### 3.3. Moxibustion Time in the Experimental Group

Different from the control group, moxibustion dose was individual in the experimental group. According the record of individual moxibustion time, the dose differed in the terms of patients' conditions and moxibustion sensation, which had been measured about 28~65 minutes in the treatment of KOA. The range of mean moxibustion dose was about 47.30 ± 6.20 minutes in the experimental group.

### 3.4. Safety

No adverse events were reported for the 72 participants.

## 4. Discussions

The results of our study extended those previous trials and demonstrated that patients with KOA who received individual eliminate-sensitive dose had significantly less pain and better function after 1 month than did patients who received stable conventional dose, according the total GPCRND-KOA score.

After 7 months, exploratory analysis indicated that the differences between the two groups were still significant. Significant differences were also evident for knee circumference.

The side-effects of heat-sensitive moxibustion were not observed. Several large surveys have also provided evidence that moxibustion is a relatively safe treatment [12–14]. To our knowledge, our study is the largest reported randomized controlled trial in this regard. Its strengths included interventions based on expert consensus by qualified and experienced medical acupuncturists, outcome measurements as recommended in guidelines for trials on KOA, and very high follow-up rates. Our study used central randomization to ensure adequate concealment in group assignment. However, one could argue that our results might have been biased by a lack of sufficient blinding. Due to the nature of the intervention, it was not possible to blind therapists to treatment. Both the evaluation of the results and the statistical analysis were carried out in a blind fashion.

Our results lent support to the findings of two previous smaller trials, one of which were randomized [5] and one was not [6]. The differences in findings might be due to low statistical power in the early trials, use of different measurement instruments and a short-term followup.

Traditionally, different moxibustion techniques were used and suspended moxibustion is the main therapy resulting from the burning of moxa produces the radiant heat and drug effects to acupoints. Heat-sensitive moxibustion is different from other conventional suspended moxibustion treatments. Generally speaking, the conventional suspended moxibustion manipulates a moxa stick in which the moxa stick is placed 2-3 cm from the skin with the intention of mildly warming local sites. Facing different patients, it selects a series of identical and fixed acupoints to treat. And the moxa stick isusually lighted around the acupoints to bring mild warmth to the area without burning, until the skin becomes slightly red. In this traditional mode, therapists consider that moxibustion can take effect whenthe patients feel local and surface heat sensation. Time consumed in this regimen is identical length for various patients. Namely, moxibustion time per acupoint is uniform but not individual. We believe that the potential clinical efficacy of moxibustion cannot be produced by conventional suspended moxibustion treatment. A number of evidences proved heat-sensitive moxibustion were superior to needle acupuncture and conventional suspended moxibustion in clinical practice [2]. The reason is that moxibustion dose in heat-sensitive moxibustion is different from that in conventional suspended moxibustion.

Theoretically, three factors have an impact on moxibustion dose, including intensity, area, and time. The two former factors are routine as a result of the standard size of moxa stick in practice. Hence, time becomes a variable parameter and plays a quite important part in moxibustion dose. We wonder how long the optimum dose should be obeyed in heat-sensitive moxibustion. The simplest method refers to the stable 15 minutes, which was recommended by the universal text book. In the book of Method of Needling and Moxibustion, which was considered as authoritative teaching material in moxibustion technique in China [1], a practitioner should light one end of a moxa stick and hold it an inch or two away from the skin, usually around the inserted needles to bring mild warmth to the area without burning, until the skin becomes slightly red. The moxibustion is recommended as 15 minutes in this book. Practitioners use moxa to warm regions and acupuncture points with the intention of stimulating circulation through the points and inducing a smoother flow of blood and qi. It is claimed that moxibustion takes, effect in the body with 15 minutes moxibustion.

In this study, our result supported that the individual eliminate-sensitive dose was likely to become an optimum selected regimen in heat-sensitive moxibustion. The amount of moxibustion per acupoint was not fixed, according to the patient condition and moxibustion sensation. Treatment sessions ended when patients felt the acupoint heat-sensitization phenomenon disappeared. Thus, the dose is individual and fit for every patient. It reflects the requirement from patients' bodies. We termed it as individual eliminate-sensitive dose. In our study, the range of mean moixbustion dose was about 47.30 ± 6.20 minutes in the experimental group. The maximum length was 65 minutes, while the shortest one was 28 minutes. As far as we know, the feasibility of such a time intensive treatment can be carried out by normal acupuncture-practitioner's office in China. Many TCM hospitals possessed qualified specialized moxibustion technicians. Meanwhile, various moxibustion devices were used to fix the moxa-sticks and reduced manual labour.

As we all know, KOA is an incurable complaint of musculoskeletal system disease. The aim of initial design was to explore a better dose of heat-sensitive moxibustion in the treatment of KOA. Special theory penetrated in our thought and design, which is the presupposition and basis in this trial. In the human nature, there are two states of acupoints, the stimulated or awake state and the rest state. When the human body suffers form disease, the acupoints on the surface of the body are stimulated and sensitized. These sensitized acupoints are changed into rest ones in the course of treatment. The elimination of heat-sensitization phenomenon is the indication to examine clinical efficacy. Our final results confirmed the theory involving with heat-sensitive moxibustion to some extent. We also found an approach to quantify moxibustion dose individually in clinical practice.

In a word, the result demonstrated that the effectiveness of individual eliminate-sensitive dose might be more superior to the stable conventional dose in the treatment of KOA. Obviously, only 15 minutes in the moxibustion dose was very difficult to show and uncover the real clinical effects of suspend moxibustion therapy. According the record of individual moxibustion time, the dose differed in the terms of patients' conditions and moxibustion sensation, which had been measured about 28~65 minutes in the treatment of KOA. In future, if we can confirm this conclusion in other diseases. Through more rigorous and scientific evidences, it will be helpful to disclose inherent law of moxibustion.

##  Conflict of Interests

The authors declare that they have no competing interests.

##  Authors' Contribution

R. Chen and, M. Chen obtained funding for the research project. J. Xiong, Z. Chi, and B. Zhang drafted the protocol, and J. Xiong wrote the final paper. Z. Chi, S. Tongsheng, S. Jianhua, and Z. Meiqi contributed to the research design and made critical revisions. B. Zhang was responsible for the statistical design of the trial and wrote portions of the statistical methods, data handling, and monitoring sections. All authors read and approved the final paper.

## Figures and Tables

**Table 1:** Baseline characteristics of KOA patients.

	Individual group	Stable group
Age, mean (SD), years	58 (8.03)	59 (6.94)

Age, min-max, years		
Age, min-max, years	42~70	45~70
Age >60 year *n* (%)	14 (38.89%)	12 (33.33%)

Sex *n* (%)		
Male	8 (22.22%)	6 (16.67%)
Female	28 (77.78%)	30 (83.33%)

Duration of knee pain *n* (%)		
<5 year	24 (66.67%)	30 (83.33%)
5–10 year	4 (11.11%)	3 (8.33%)
>10 year	8 (22.22%)	3 (8.33%)

BMI, mean (SD), kg/m′	23.08 (2.71)	21.52 (2.43)

BMI, min-max, kg/m′	15.64~31.08	14.98~28.11

GPCRND-KOA grade *n* (%)		
Severe	24 (67%)	21 (58%)
Moderate	12 (33%)	15 (42%)

Knee circumference at baseline(SD), cm	39.32 (3.43)	39.01 (2.41)

GPCRND-KOA score at baseline		
Total score mean (SD)	11.22 (3.06)	10.14 (3.00)
Pain or discomfort in night score mean (SD)	1.33 (0.68)	1.25 (0.73)
Morning stiffness score mean (SD)	0.97 (0.56)	1.16 (0.51)
Pain or discomfort in walk score mean (SD)	1.67 (0.53)	1.55 (0.64)
Arise from seat score mean (SD)	0.64 (0.49)	0.58 (0.50)
The maximum walk distance score mean (SD)	1.86 (0.90)	1.50 (0.85)
Board standard airstairs score mean (SD)	1.08 (0.55)	1.14 (0.35)
Step down standard airstairs score mean (SD)	1.19 (0.52)	1.05 (0.41)
Squat or bend knees score mean (SD)	1.36 (0.59)	1.17 (0.61)
Walk over rough terrain score mean (SD)	1.06 (0.53)	0.86 (0.42)

Previous treatment (past-half year)		
Pharmaceutical intervention	18 (50.00%)	16 (44.00%)
Physiotherapy	7 (19.44%)	8 (22.22%)
Previous acupuncture treatment	2 (5.56%)	3 (8.33%)

BMI: body mass index; GPCRND-KOA: guiding principle of clinical research on new drugs in the treatment of KOA score; SD: standard deviation; KOA: knee osteoarthritis.

**Table 2:** Comparison of GPCRND-KOA scores at baseline, end of treatment (month 1), and followup (month 7), together with between-group statistical test.

Variable	Baseline			Month 1			Month 7		
	Mean	SD	*t*-test*	Mean	SD	*t*-test*	Mean	SD	*t*-test*
Total									
Individual group	11.22	3.06	1.51	3.44	1.93	4.51	2.19	1.85	6.31
	95% CI = (10.50~11.94)			95% CI = (2.99~3.89)			95% CI = (1.76~2.62)		

Stable group	10.14	3.00		6.13	3.01		5.11	2.07	
	95% CI = (9.44~10.84)			95% CI = (5.42~6.84)			95% CI = (4.62~5.60)		

*t-test of group differences at time period, using independent *t*-test. All data are intention to treat. Both groups *n* = 36. SD: standard deviation; GPCRND-KOA: guiding principle of clinical research on new drugs in the treatment of KOA score; KOA: knee osteoarthritis.

**Table 3:** Comparison of Knee circumference scores at baseline, end of treatment (month 1), and followup (month 7), together with between-group statistical test.

Variable	Baseline			Month 1			Month 7		
	Mean	SD	*t*-test*	Mean	SD	*t*-test*	Mean	SD	*t*-test*
Knee circumference, cm									
Individual group	39.32	33.42	0.44	36.21	3.40	2.51	35.81	3.63	2.78
	95% CI = (38.52~40.12)			95% CI = (35.41~37.01)			95% CI = (34.96~36.66)		

Stable group	39.01	2.41		38.03	2.72		37.92	2.74	
	95% CI = (38.44~39.58)			95% CI = (37.39~38.67)			95% CI = (37.28~38.56)		

*t-test of group differences at time period, using independent *t*-test. All data are intention to treat. Both groups *n* = 36. SD: standard deviation.
